# p27/Kip1 functions as a tumor suppressor and oncoprotein in osteosarcoma

**DOI:** 10.1038/s41598-019-42450-0

**Published:** 2019-04-16

**Authors:** Arthur W. Currier, E. A. Kolb, Richard G. Gorlick, Michael E. Roth, Vidya Gopalakrishnan, Valerie B. Sampson

**Affiliations:** 1 Nemours Biomedical Research, Nemours/Alfred I. duPont Hospital for Children, Wilmington, DE 19803 USA; 2 University of Delaware, Department of Biological Sciences, Newark, DE 19716 USA; 3 Division of Pediatrics, University of Texas MD Anderson Cancer Center, Houston, TX 77030 USA

## Abstract

The p27/kip1 (p27) tumor suppressor inhibits cyclin/cyclin-dependent kinase (CDK) complexes and halts cell cycle progression. p27 further regulates invasion and migration in cancer cells, suggesting p27 also functions as an oncoprotein. Using a human osteosarcoma tissue microarray we identified high expression of cytoplasmic p27 in metastatic tumors. We demonstrated a positive correlation between mRNA and protein expression of p27 and expression of key metastatic markers, vimentin, snail-2, β-catenin and stathmin-1 (STMN1) in patient tumors. Our results show that T198 phosphorylation of p27 controls the interaction between p27 and STMN1 that regulates microtubule stabilization and the invasion and migration of osteosarcoma cells. We found that anti-tumoral activity of gemcitabine and the Wee1 kinase inhibitor AZD1775 in osteosarcoma cells, was dependent on drug sequencing that relied on p27 stabilization. Gemcitabine activated caspase-3 and synergized with AZD1775 through caspase-mediated cleavage of p27, that dissociated from STMN1 and effectively induced apoptosis. Further, blockage of nuclear export of p27 by inhibition of Exportin-1 (XPO1) promoted growth arrest, demonstrating that the biological effects of agents relied on the expression and localization of p27. Together, these data provide a rationale for combining chemotherapy with agents that promote p27 tumor suppressor activity for the treatment of osteosarcoma.

## Introduction

Osteosarcoma is the most common bone malignancy that affects primarily children and young adults. Increasing our understanding of the complex biology of osteosarcoma tumors and how tumors evolve will provide opportunities to improve outcomes for patients who present with metastases and those at-risk for metastatic progression. The p27(Kip1) protein (encoded by *CDKN1B*), participates in many signal transduction pathways and mediates tumor suppressor or oncogenic roles in cancer. Understanding the functions of p27 in osteosarcoma could provide leads for developing novel effective therapeutic strategies.

The p27 protein inhibits the activity of cyclin dependent kinase (CDK) 2–cyclin E and CDK4–cyclin D complexes at the G1-phase and functions as a CDK-inhibitor that halts the S-phase transition^1,2^. Mitogenic signals that decrease p27 expression allow CDK/cyclin activation and cell cycle progression^3^. However, over the past decade many studies have characterized roles of p27 in signaling pathways that affect cell proliferation, survival, differentiation and migration^4^. These are often described as non-canonical p27 functions since they are independent of CDK/cyclin regulation. A central feature in describing the non-canonical activity of p27 is its cytoplasmic localization. Cytoplasmic p27 participates in cellular processes including cytoskeleton dynamics, cell migration and metastasis^5,6^. This is consistent with reports that cytoplasmic p27 plays a role in processes that are associated with tumor development and disease progression.

Phosphorylation of several binding domains of p27 regulates its expression, localization, and interaction with other proteins. The CDK inhibitory domain of p27 resides in the N-terminal region and consists of Serine 10 (S10) and amino acids 20–90^7^. This domain contains an α-helix that binds to CDK and cyclin subdomains and exhibits a broad specificity toward CDK2, CDK4 and CDK6^1,2^. In mammalian cells, phosphorylation of S10 in the N-terminus by mitogen-activated protein kinase (MAPK)^8^, and phosphatylinositol-3-kinase (PI3K)/Akt1 signaling^9^ is required for nuclear export of p27 by exportin 1 (XPO1), also known as chromosome region maintenance 1 (CRM1)^10^. Kossatz *et al*.^11^, also showed that T198 phosphorylation prevents p27 ubiquitylation and proteosomal turnover and controls protein stability. CDK2/cyclin E phosphorylates p27 at threonine-187 (T187) in late G1-phase. This allows the binding of p27 to Skp2 (S-phase kinase-associated protein 2), the F-box protein component of an SCF (SKP1-CUL1-F-box protein) ubiquitin ligase (E3) complex, for ubiquitination, and subsequent proteasomal degradation^12,13^.

Although studies have shown p27 plays a role in the dedifferentiation of osteoblasts^14^ and accumulation of p27 autoantibody in osteosarcoma patient serum samples is prognostically significant^15^, little is known about the molecular mechanisms by which p27 regulates osteosarcoma cell survival. In this study, we used human cell lines and patient tumors to examine p27 protein and mRNA expression in osteosarcoma. We compared the expression of p27 in osteosarcoma tumors with expression levels of key metastatic markers to identify a correlation with metastatic disease and explored p27 responses to the treatment of osteosarcoma cells with gemcitabine and AZD1775. This work provides insight into mechanisms involving p27 that may predict sensitivity of osteosarcoma tumors to drugs and guide treatment with targeted therapies.

## Results

### The p27 protein is localized in the cytoplasm in osteosarcoma patient tumors and cell lines

**Figure 1:**
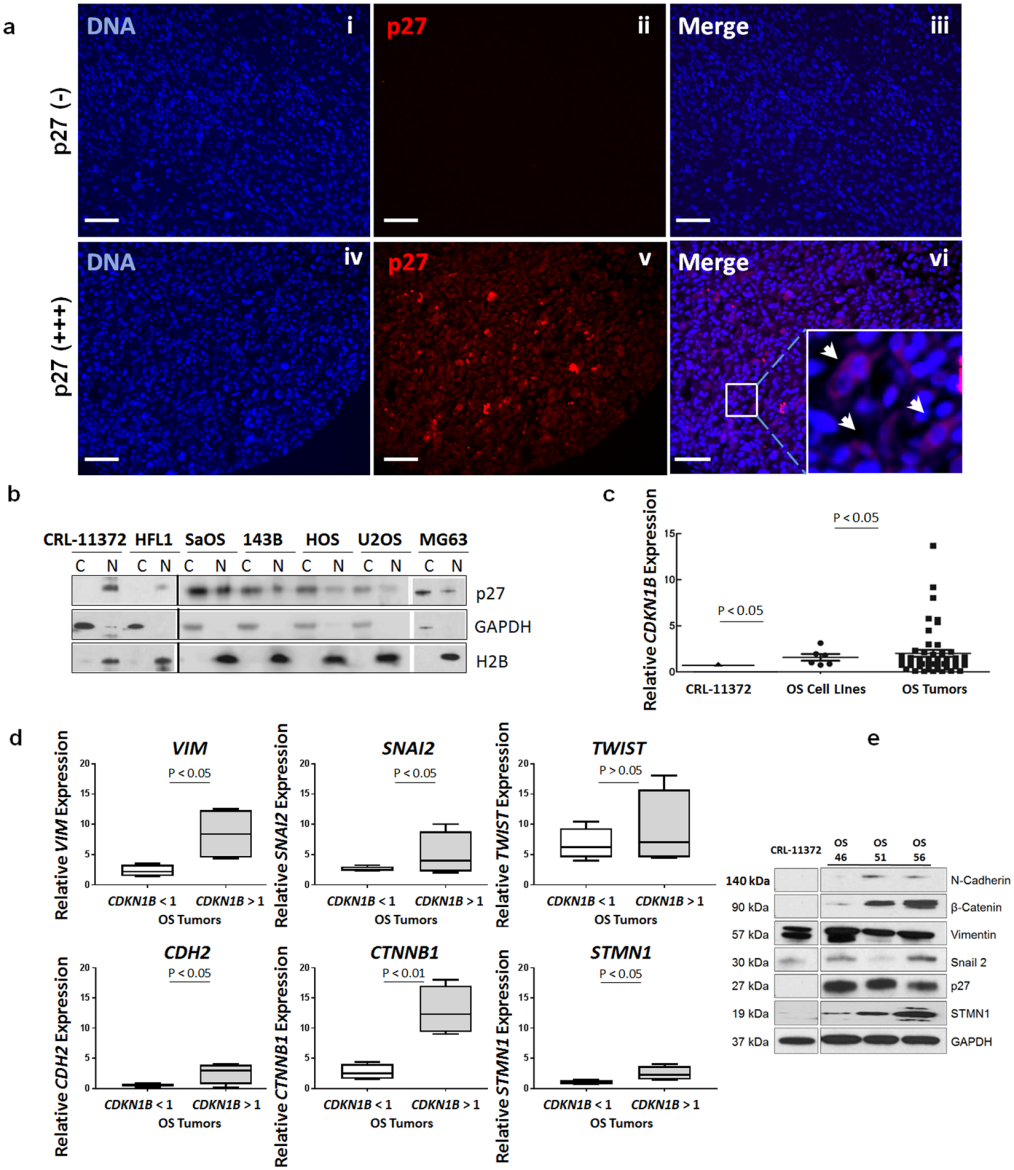
p27 protein is expressed in the cytoplasm of osteosarcoma patient tumors and cell lines. (**a**) Osteosarcoma tumors on a human osteosarcoma tissue array were subjected to immunofluorescence staining using anti-p27 antibody. Representative images are shown for tumors with negative [p27 (−)] staining, panels i-iii and tumors with positive [p27 (+)] staining, panels iv-vi. p27 is colored red; nuclei were stained with Hoechst dye and are colored blue. Insert: Amplification of boxed area shows cytoplasmic p27 (white arrows). Magnification is 20x; scale bar = 20 μm. (**b**) Nuclear and cytoplasmic lysates were extracted from osteoblasts (CRL-11372), lung fibroblasts (HFL1) and SaOS, 143B, HOS, U2OS, MG63 human osteosarcoma cell lines. Lysates were examined by immunoblot analysis with anti-p27 antibody. GAPDH was the cytosolic marker and H2B was the nuclear marker. (**c**) Total mRNA was extracted from the 5 osteosarcoma cell lines and 50 osteosarcoma patient tumors and *CDKN1B* mRNA levels were measured by RT-qPCR. mRNA expression was quantified relative to control osteoblasts, CRL-11372 (RQ = 1). Each dot (•) represents one cell line, each square (▪) represents one patient sample. Bars represent mean with standard deviation, Statistical significance is shown by p < 0.05. (**D**) The 50 osteosarcoma patient tumors were divided into two groups of low and high *CDKN1B* expressing tumors. Left box plot of each graph represents gene expression for a tumors expressing *CDKN1B* < 1 (low), right box plot represents tumors expressing high *CDKN1B* > 1 (high). Statistical significance was measured by a two-tailed t-test. (**e**) Tumor lysates were prepared from 3 patient-derived osteosarcoma xenografts and CRL-11372 osteoblasts and subjected to immunoblot analysis with antibodies against N-Cadherin, β-catenin, vimentin, Snail-2, p27 and STMN1. GAPDH was the protein loading control.

To evaluate cytoplasmic p27 expression in osteosarcoma patient tumors, we performed immunofluorescence staining of tumor specimens on a previously described tissue microarray (TMA)^16^ using anti-p27 antibody. Briefly, the TMA consisted of human osteosarcoma tumors collected at biopsy (biopsy specimens), during definitive resection of the tumor (definitive surgery specimens), and during resection of distant metastasis to the lung (metastatic tumor specimens). All patients were treated with standard MAP (methotrexate, doxorubicin, cisplatin) chemotherapy backbone. Biopsy specimens were taken before chemotherapy was given to patients, while definitive surgery and metastatic tumor specimens were taken from patients who had already been exposed to chemotherapy. Analysis was conducted for 9 biopsy, 29 definitive surgery and 14 metastatic tumor specimens (i.e. 52 evaluable osteosarcoma tumors). The variations in the intensity of p27 staining among tumors were scored as negative (−), low (+), medium (++) and high (+++) expression. Figure 1a demonstrates representative staining intensities for tumors with negative and high levels of cytoplasmic p27. A greater proportion of metastatic tissue expressed p27 compared to initial biopsy specimens (86.7% vs 45.4%, p = 0.04), Table 1. Very high expression (+++) was more prevalent in the metastatic tissue compared with both the biopsy and definitive surgery specimens (33.3% vs 5.0%, p = 0.01). These results suggest that there was a correlation between high expression of cytoplasmic p27 and metastatic disease. The localization of p27 protein was also examined in 5 human osteosarcoma cell lines (SaOS, 143B, HOS, U20S, MG-63), normal osteoblasts (CRL-11372) and lung fibroblasts (HFL1). Cytoplasmic (C) and nuclear (N) lysates were extracted and assessed by immunoblot analysis. As shown in Fig. 1b (Supplementary Fig. S1), p27 was detected in the cytoplasm and nucleus of osteosarcoma cells, but was primarily localized in the nucleus of osteoblasts and fibroblasts. These results are consistent with the stabilization and cytoplasmic localization of p27 that is seen in human tumors and cancer cells that express p27^17^.

**Table 1:** Immunofluorescence analysis of cytoplasmic p27 expression in osteosarcoma tumors.

Score	Biopsy (n = 11)	Definitive Surgery (n = 29)	Metastatic Tumor (n = 15)
p27-Negative	6 (55%)	10 (34%)	2 (14%)
+	1 (9%)	8 (28%)	3 (20%)
++	4 (36%)	9 (31%)	5 (33%)
+++	—	2 (7%)	5 (33%)

p27 cytoplasmic expression was scored as negative or positive (**+**/weak, **++**/medium, **+++**/strong).

We also determined the expression of *CDKN1B* mRNA by RT-qPCR using RNA extracted from the 5 osteosarcoma tumor cell lines and 50 patient osteosarcoma tumors. The relative quantity (RQ) of tumor mRNA was normalized to osteoblasts (RQ = 1). As shown in Figure 1c, the mean RQ value of osteosarcoma cell lines was 2.0 (p < 0.05) and the mean value for the patient tumors was 2.2 (p < 0.05), in comparison to osteoblasts. To determine whether there was a correlation of *CDKN1B* expression with mRNA levels of known metastatic genes, we further measured mRNA expression of vimentin (*VIM*), snail-2/slug (*SNAI2*), twist (*TWIST*), N-cadherin (*CDH2*), β-catenin (*CTNNB1*), and stathmin-1 (*STMN1*) using the 50 patient tumors. Data is grouped for low mRNA expressing tumors (*CDKN1B* < 1, n = 22 specimens) and high mRNA expressing tumors (*CDKN1B* > 1, n = 28 specimens). These results show a positive correlation for 5 metastatic markers, *VIM*, *SNAI2*, *CDH2*, *CTNNB1* and *STMN1*, Fig. 1d, but an association was not observed for *TWIST* (p > 0.05) in the tumors.

We further explored whether high protein expression of p27 protein correlated with the protein levels of metastatic markers. We examined tumor cell lysates prepared from 3 patient-derived xenograft (PDX) tumors expressing high levels of p27 by immunoblot analysis, using antibodies against vimentin, snail-2, N-cadherin, β-catenin and STMN1. As shown in Fig. 1e (Supplementary Fig. S2) expression levels of the metastatic markers were upregulated in PDX tumors, in comparison to osteoblasts. Collectively, these data demonstrate that high mRNA and protein expression of p27 as well as localization to the cytoplasm in osteosarcoma tumors are associated with metastatic disease.

### Phosphorylation at T198 controls the interaction between p27 and STMN1 and regulates p27 cytoplasmic function

**Figure 2:**
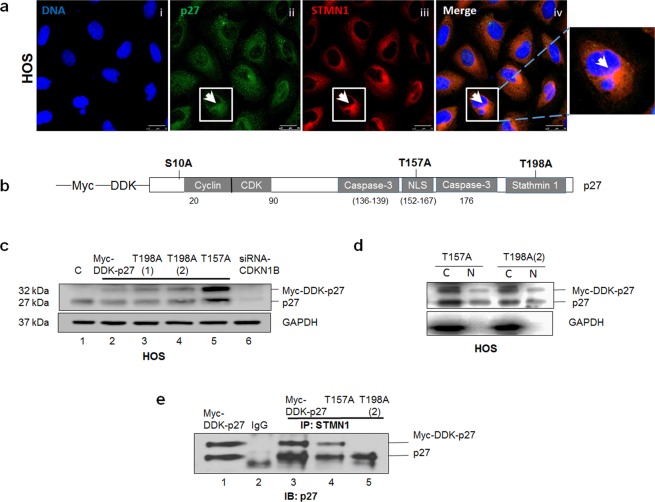
The interaction between p27 and STMN1 is dependent on T198 phosphorylation. (**a**) HOS cells were subjected to immunofluorescence staining using anti-p27 and anti-STMN1 antibodies. p27 is colored green; STMN1 is colored red; nuclei were stained with Hoechst dye and are colored blue. Amplification of boxed area highlights the merged staining of cytoplasmic p27 and STMN1 for a single cell. Magnification is 63x; scale bars = 25 μm. (**b**) Schematic representation of selected phosphorylation sites (S10, T157, T198), protein binding domains (cyclin/CDK, caspase-3, stathmin1) and the nuclear localization sequence (NLS) within the p27 amino acid sequence. (**c**) HOS cells expressing recombinant wild-type p27 (Myc-DDK-p27), T157A and T198A mutant p27 protein, and attenuated p27 (siRNA*CDKN1B)* were lysed and examined by immunoblot analysis with anti-p27 antibody. Recombinant wt p27 protein is represented at 32 kDa and endogenous p27 is at 27 kDa. GAPDH was the protein loading control. (**d**) Nuclear and cytoplasmic lysates were extracted from HOS cells expressing T157A and T198A mutant p27 protein. Lysates were examined by immunoblot analysis with anti-p27 antibody. GAPDH was the cytosolic marker. (**e**) HOS cells expressing Myc-DDK-p27, T157A and T198A mutant p27 protein were lysed and subjected to immunoprecipitation with anti-STMN1 antibody and p27 detection with anti-p27 antibody. The results are representative of three independent experiments.

Since our current data suggest that tumors with high expression levels of p27 and STMN1 show increased metastatic potential, we analyzed the interaction between these two proteins. Strong cytoplasmic staining of p27 and STMN1 in HOS cells was observed by immunofluorescence analysis, Fig. 2a. Several studies have reported that phosphorylation at S10, T157 and T198 amino acids targets p27 to the cytoplasm^9^ and T198 phosphorylation can affect STMN1 binding^18^, (illustrated in the schematic in Fig. 2b). To study the interaction between p27 and STMN1, we used the pCMV6-Myc-DDK tagged vector containing the *CDKN1B* codon to generate T157A and T198A p27 point mutations (Supplementary Fig. S3). HOS cells were transfected with either wild type (wt) or mutant plasmids and the steady-state protein levels were assessed. The immunoblot shows that expression of recombinant wt, T157A and T198A mutant p27 protein was detected at 32 kDa and endogenous p27 was also detected at 27 kDa, Fig. 2c (Supplementary Fig. S3). We confirmed these two bands by p27 depletion with siRNA*CDKN1B* targeting oligonucleotides, (Fig. 2c, Lane 6). We also show that cells expressing T157A or T198A protein exhibited cytoplasmic and nuclear localization of mutant p27 protein, (Fig. 2d, Supplementary Figure S4).

To test whether p27 was bound to STMN1, an immunoprecipitation assay was performed with cell lysates extracted from asynchronously growing HOS cells stably expressing either wt p27, T157A or T198A mutant p27 protein, using anti-STMN1 antibody. Immunoblot analysis with anti-p27 antibody revealed recombinant wt p27 and endogenous p27 were bound to STMN1 and a weaker association was evident for T157A p27 protein (Fig. 2e, lanes 3 and 4, Supplementary Fig. S5). However, the T198A mutant protein was not detected (Fig. 2d, lane 4), indicating that the T198A mutation prevented interaction with STMN1. These results demonstrate that the p27 and STMN1 interaction is directly dependent on phosphorylation at T198. Of note, previous studies^11^ showed that the T198A mutation stabilizes nuclear p27, that leads to protein degradation. This suggests that delayed nuclear export may in part, lower levels of cytoplasmic T198A p27 and indirectly contribute to loss of STMN1 binding (Fig. 2e).

### p27 and STMN1 interaction regulates cytoskeletal dynamics, invasion and migration of osteosarcoma cells

**Figure 3:**
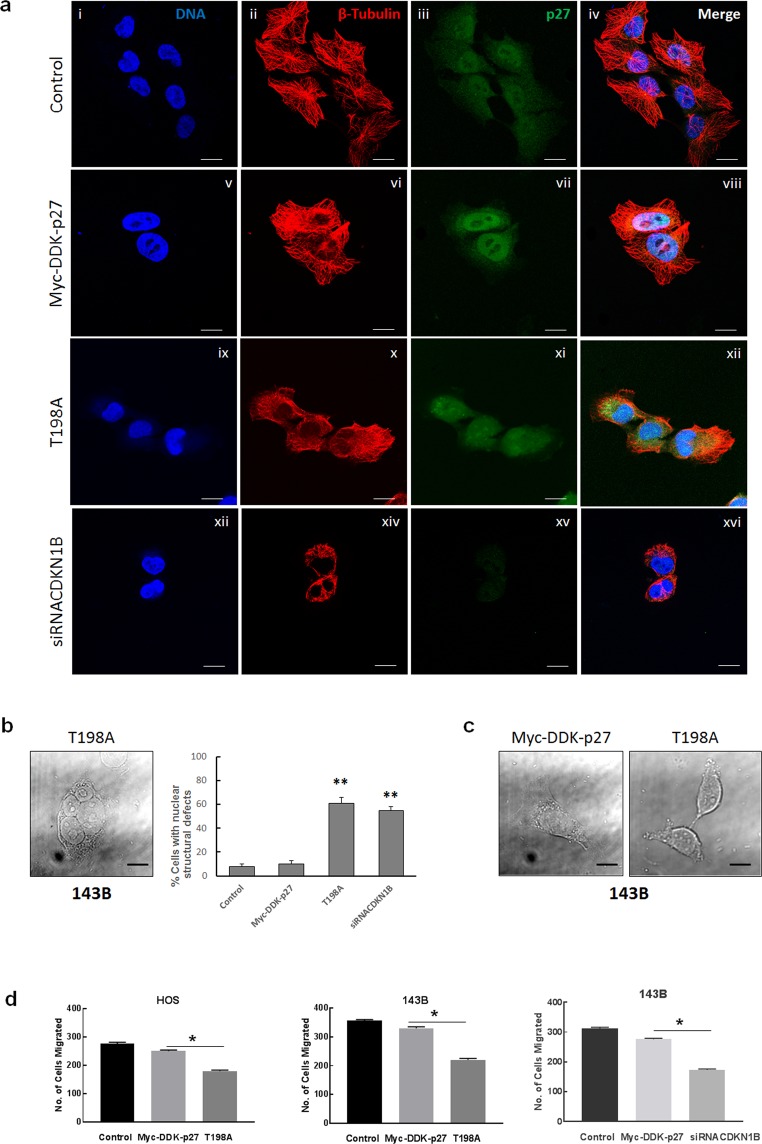
p27 and STMN1 interaction regulates microtubule stabilization and migration of osteosarcoma cells. (**a**) 143B untransfected controls and cells expressing recombinant wild-type p27 (Myc-DDK-p27), T198A mutant p27 (T198A) and p27 depletion were subjected to immunofluorescence staining with antibodies against β-tubulin and p27. β-tubulin is colored red; p27 is colored green; nuclei were stained with Hoechst dye and are colored blue. Magnification is 63x; scale bars = 25 μm. (**b**) 143B cells were imaged using differential interference contrast (DIC) microscopy and analyzed for nuclear structural defects. Left panel: Representative image of a polynucleated cell expressing T198A mutant p27. Right plot: Each column represents percentage of nuclear structural defects for 100 cells for indicated experimental groups. *p < 0.5. (**c**) DIC image of 143B control cell and apoptotic cell expressing T198A mutant p27. Magnification is 63x; scale bars = 25 μm. (**d**) The matrigel invasion assay was conducted using HOS (left plot) and 143B cells (middle and right plots) expressing recombinant wild-type p27, T198A mutant p27 and reduced p27 (siRNA*CDKN1B*). Data is shown as mean + SE from three experiments; *represents p < 0.05.

Since STMN1 is a critical regulator of microtubule dynamics, we next investigated whether loss of the ability of p27 to bind STMN1 affected cytoskeletal organization. Asynchronous 143B cells expressing either recombinant wt or T198A mutant p27, or attenuated p27 protein (by siRNA*CDKN1B*), were evaluated by immunofluorescence staining using anti-p27 and anti-β-tubulin antibodies. An intact microtubule cytoskeleton was observed for cells expressing recombinant wt p27 that showed increases in cytoplasmic and nuclear p27 staining (Fig. 3a, panels v-viii). This was similar to untransfected control cells (panels i-iv), and cells transfected with empty pCMV-Myc-DDK vector (Supplementary Fig. S6). Cells expressing T198A mutant p27 protein also showed increases in cytoplasmic and nuclear p27 staining, but in contrast, significant disruption of microtubules was evident, (panels ix-xii). Destabilization of microtubules was also seen for cells with p27 depletion (panels xiii-xvi, Supplementary Fig. S7). Additional analysis was performed to determine the nuclear structure of these cells. Cells expressing either T198A p27 protein or attenuated p27 were examined by DIC microscopy. Figure 5b shows the development of nuclear defects, including enlarged nuclei, binuclei, polynuclei and micronuclei. The percent of nuclear structural defects was scored relative to controls and are shown in Fig. 3b, right plot. Additionally, cells with cytosol condensation and membrane blebbing were also observed, Fig. 3c. Together, these results indicate that T198A mutant p27 protein promotes destabilization of microtubules, mitotic catastrophe and can induce apoptosis in osteosarcoma cells.

To investigate the effect of the loss of interaction between p27 and STMN1 on the mobility of osteosarcoma cells, we used HOS and 143B cell lines expressing either recombinant wt or T198A mutant protein to perform migration and invasion assays. The number of cells expressing mutant p27 that migrated through matrigel was significantly lower than control cells expressing recombinant wt protein (approximately 50% for HOS and 55% for 143B), Fig. 3d (left plot). We also compared the migration of 143B cells expressing recombinant wt protein and cells with depleted p27 protein, relative to control cells transfected with non-targeting siRNA. As shown in Fig. 3d (right plot), a significant reduction in migration was found for cells with p27 depletion, and this result was similar to that observed for cells expressing T198A mutant protein. Collectively, our data revealed that T198 phosphorylation regulates p27 activity through the interaction with STMN1 that stabilizes the microtubule cytoskeleton and promotes osteosarcoma cell migration.

### Cell cycle inhibition therapy potentiates the effect of chemotherapy *in vitro* through upregulation and cleavage of p27

**Figure 4:**
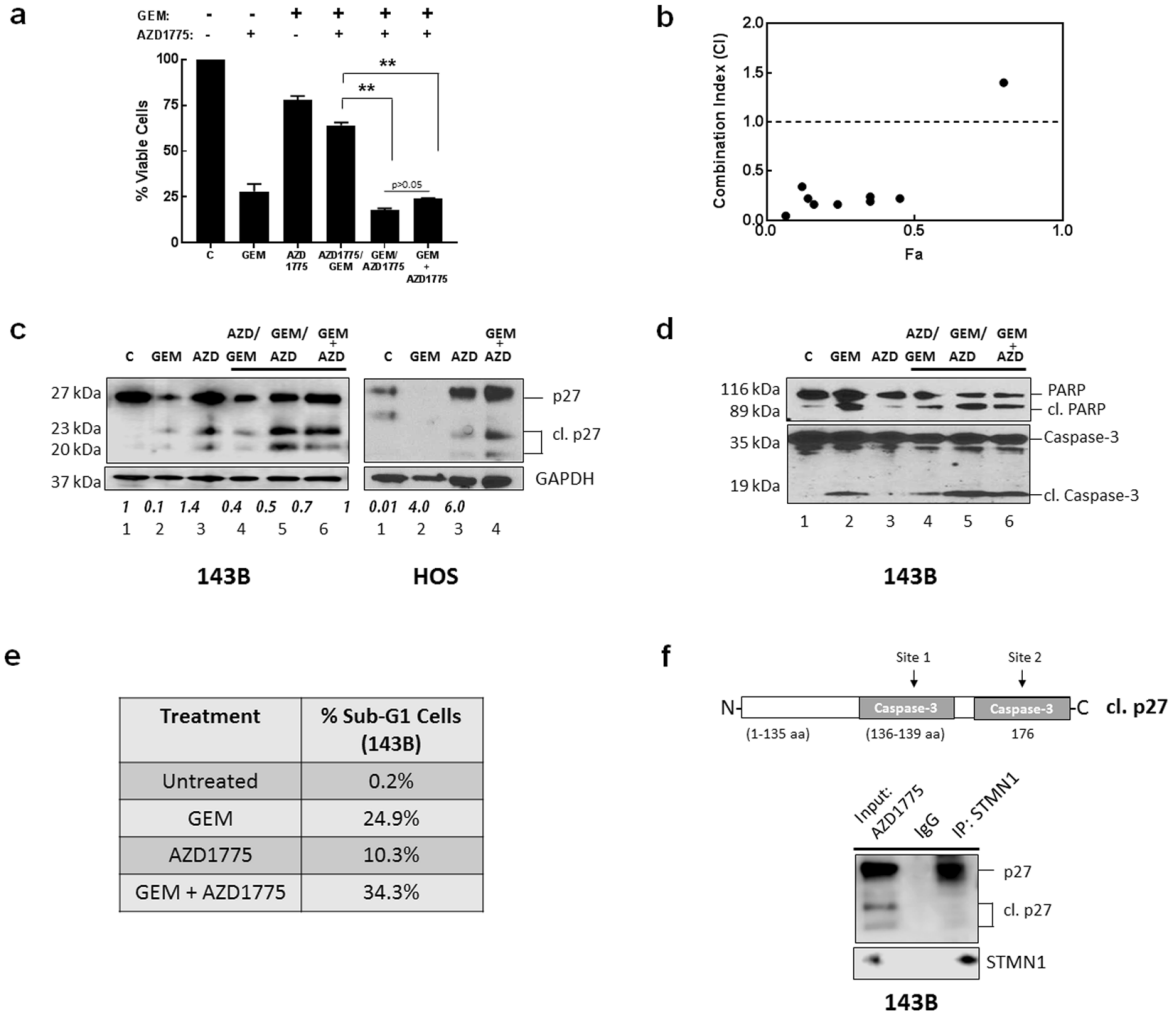
AZD1775 causes accumulation of p27 form in osteosarcoma cells. **(a**) 143B cells were incubated with gemcitabine (GEM), AZD1775 or the indicated combinations of both agents and cell viability was measured using the cell titer blue assay. Data is shown as mean + SE; *represents p < 0.05; n = 6. (**b**) Chou-Talalay analysis of synergy in 143B cell lines for GEM/AZD1775 treatment. CI < 1 indicates synergy of agents. Drug dosing concentrations are listed in Supplementary Table S1. (**c**) Immunoblot analysis of lysates extracted from 143B cells treated with GEM and AZD1775 as indicated using anti- p27 antibody. GAPDH was the protein loading control. Relative expression of cleaved p27 (23 kDa) was quantified from immunoblots using Image J. Results shown are mean + SE from three experiments. (**d**) Immunoblot analysis of the apoptotic markers cleaved PARP and cleaved caspase-3 were examined by immunoblot analysis using cell lysates isolated from cells exposed to the indicated treatments with GEM and AZD1775. (**e**) The cell cycle distribution was analyzed by flow cytometry using propidium iodide DNA staining following the indicated treatments of cells with GEM and AZD1775. The percent of sub-G1 cells is listed. The results are representative of three independent experiments. **(f**) HOS cells treated with AZD1775 were lysed and subjected to immunoprecipitation with anti-STMN1 antibody; p27 was detected by immunoblot with anti-p27 antibody (top panel) and STMN1 was detected with anti-STMN1 antibody (lower panel).

In a previous study, gemcitabine and AZD1775 (Wee 1 kinase inhibitor) demonstrated efficacy against osteosarcoma xenograft tumors^19^. We determined the effects of gemcitabine and AZD1775 on human osteosarcoma cell growth *in vitro* and examined whether the efficacy of agents was dependent on p27 status. Asynchronously growing HOS and 143B cells were treated with gemcitabine or AZD1775, either as single agents or combined. Combination treatments were the sequential administration of agents, comprising of incubation with each drug for 24 h and co-treatment with both drugs for 24 h, to enable comparisons for 24 h exposure to each agent (see Methods). As shown in Fig. 4a, when drugs were administered as single agents, 143B cells showed higher sensitivity to gemcitabine in comparison to AZD1775 (30% vs 78% viable cells, respectively). When the two drugs were combined, highest sensitivity was observed for gemcitabine followed by AZD1775 (GEM/AZD1775) and co-treatment (GEM + AZD1775), resulting in 18% and 22% viable cells, respectively, (p > 0.05). In contrast, cells treated with AZD1775 followed by gemcitabine (AZD1775/GEM) showed decreased sensitivity to agents, resulting in 65% viable cells (p < 0.05). HOS cells were also sensitive to drug treatment in a similar manner, (Supplementary Fig. S8). The Combination Index (CI) values for the sequential treatment of GEM/AZD1775 were calculated from dose-response curves by the Chou and Talalay method and are shown in Fig. 4b and Supplementary Table S1. CI values were <1 and validate the synergy of agents.

Since these results demonstrated that drug sequences have significantly different levels of activity, we analyzed the effects on the status of p27 to determine antitumor effects of agents. Cell lysates for all treatment groups were examined by immunoblot analysis. Figure 4c (Supplementary Fig. S9) shows that cells that were sensitive to gemcitabine showed downregulation of p27 expression (lane 2) while the p27 levels increased after AZD1775 treatment (lane 3). Importantly, these results also revealed that following these drug treatments two p27 bands at 23 kDa and 15 kDa (Fig. 4c) were upregulated, in comparison to controls. Notably, other studies have demonstrated that p27 contains caspase recognition sites at D139 and D176^20,21^ and these can be cleaved to produce truncated protein isoforms at 23 kDa and 15 kDa respectively, that lack the C-terminal region (schematic representation, Fig. 2b). Apoptosis was then measured by detecting the expression of the apoptotic markers, cleaved caspase-3 and cleaved PARP in cell lysates. Figure 4d (Supplementary Fig. S10) shows there was a great activation of apoptosis for gemcitabine treatment, in comparison to AZD1775 treatment. This suggests the downregulation of p27 by gemcitabine was mediated by caspase-3 cleavage, that led to protein degradation (Fig. 4d, lane 2). Our results show that AZD1775 upregulated full length and cleaved p27 protein, although cleaved caspase-3 was not detected by immunoblot (Fig. 4d, lane 3). Further, apoptosis was detected for the three drug combinations and was highest for GEM/AZD1775 and GEM + AZD1775. These results suggest that 143B cells were sensitive to gemcitabine that activated caspase-3 and cleaved p27. This effect was potentiated by AZD1775 that upregulated p27, leading to increased cleaved p27 levels and amplified apoptosis. In contrast, pretreatment with AZD1775 upregulated p27 but attenuated gemcitabine-mediated apoptosis, that downregulated and cleaved p27, and reduced apoptosis. Together, these results suggest that greatest efficacy of drug combinations is achieved when caspase-3 activation occurs before or simultaneously with p27 upregulation.

Since cleaved p27 was observed with AZD1775 treatment, we further investigated the inhibition of 143B cell proliferation by gemcitabine and AZD1775 by flow cytometry. Apoptosis was determined by measuring the percent of 143B cells with sub-G1 DNA content. Following the exposure to gemcitabine, there were increases in the fraction of cells in the G1/S phase and the sub-G1 peak analysis showed 24.9% of apoptotic cells (Fig. 4e, Supplementary Fig. S11), in comparison to untreated control cells. The analysis for AZD1775 revealed an increase of cells in G2/M phase and a low level of apoptotic cells was detected (10.3%). These data suggest that gemcitabine inhibits 143B cell proliferation by G1/S phase cell cycle arrest, and AZD1775 causes G2/M cell cycle arrest that induces apoptosis. Also, gemcitabine exhibited a greater increase in apoptotic cells, relative to AZD1775. Analysis of the cell cycle profiles further revealed that simultaneous drug treatment of GEM + AZD1775 resulted in a greater fraction of cells in the G1/S phase and sub-G1 (34.3%) and confirmed the G1/S phase arrest of cells was mainly a result of gemcitabine, Fig. 4e.

We conducted STMN1 immunoprecipitation and immunoblot analysis, to detect bound p27 in AZD1775 cell lysates. Figure 4f (Supplementary Fig. S12) confirmed that STMN1 was bound to full-length p27 but not to the cleaved protein. Collectively, these results demonstrate that combining chemotherapy with cell cycle inhibition has different effects on induction of apoptosis and is dependent on p27 status.

### Inhibition of nuclear p27 export synergizes with cell cycle inhibition therapy and suppresses osteosarcoma cell growth

**Figure 5:**
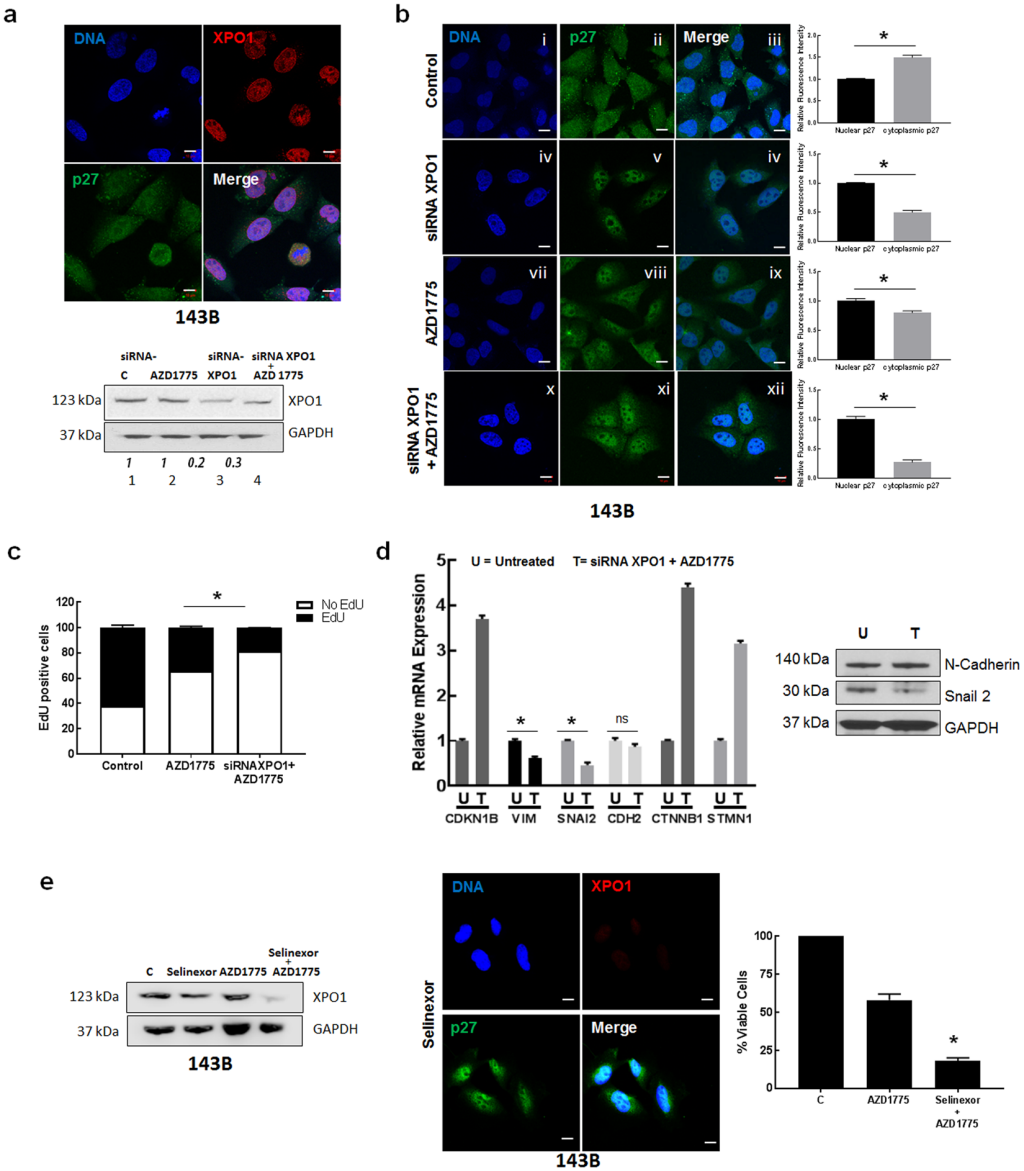
Nuclear localization and upregulation of p27 expression decrease osteosarcoma cell proliferation *in vitro*. (**a**) Upper panel: A. 143B cells were subjected to immunofluorescence staining using anti-XPO1 and anti-p27 antibodies. p27 is colored green; XPO1 is colored red; nuclei were stained with Hoechst dye and are colored blue. Magnification is 63x; scale bars = 10 μm. Lower panel: Immunoblot analysis of lysates extracted from 143B cells treated with AZD1775, siRNA*XPO1* or both agents combined with anti-XPO1 antibody. GAPDH was the loading control. (**b**) Immunofluorescence staining of 143B cells was performed with anti-p27 antibody. p27 is colored green; nuclei are stained with Hoechst dye and colored blue. The merged panel shows nuclear and cellular localization of p27. Magnification is 63x; scale bars = 10 μm. Right plots: Relative intensities of nuclear and cytoplasmic p27 staining were quantified by Image J. (**c**) 143B cells were treated as indicated and analyzed using the EdU assay. Cell proliferation plot was generated by determining EdU positive cells. Each column represents EdU positive (black) and negative (white) cells for 100 cells. Data is mean + SE; *represents p < 0.05; n = 3 replicates. (**d**) Left: Total RNA was extracted from siRNA*XPO1* + AZD1775 treated 143Bcells (T) treated cells and mRNA levels of selected metastasis genes were measured by qRT-PCR. Results are quantified relative to untreated controls (U). Statistical significance was measured by a two-sided t-test. Right: Immunoblot analysis of lysates extracted from 143B cells treated siRNA*XPO1* + AZD1775 using anti-N-Cadherin and anti-Snail-2 antibody. GAPDH was loading control. (**e**) Left: Immunoblot analysis of lysates extracted from 143B cells treated with Selinexor, AZD1775, and both agents combined (Selinexor + AZD1775). GAPDH was the loading control. Middle: Immunofluorescence staining of 143B treated with Selinexor cells was performed with anti-p27 antibody. p27 is colored green; nuclei are stained with Hoechst dye and colored blue. The merged panel shows nuclear and cellular localization of p27. Magnification is 63x; scale bars = 10 μm. Right: Cell viability of 143B cells incubated with the indicated drug treatment was measured using the cell titer blue assay. Data is shown as mean + SE; *represents p < 0.05; n = 6.

We next investigated whether changes in the localization of p27 also alter antitumor effects of therapy. Previous reports have shown that the specific inhibition of XPO1 suppresses tumor growth and represents a novel target for anticancer therapy^22^. Since overexpression of XPO1 has been implicated in osteosarcoma tumor development and progression^23^, we studied the effect of XPO1 inhibition on p27 localization. Immunofluorescence staining shown in Fig. 5a (top panel) confirmed the high expression of XPO1 in 143B cells. Cells were transfected with siRNA*XPO1* oligonucleotides and evaluated by immunoblot with anti-XPO1 antibody. Figure 5a (bottom panel and Supplementary Fig. S13) illustrates expression of XPO1 was decreased. Immunofluorescence staining with anti-p27 antibody shows increased nuclear p27 staining in cells following XPO1 depletion, in comparison to control cells transfected with non-targeting siRNA oligonucleotides, Fig. 5b (panels v and vi). We also examined the effect with AZD1775 and confirmed high expression of nuclear p27 in cells after AZD1775 treatment (panels viii and ix). Further increases in levels of nuclear p27 were observed upon exposure of siRNA*XPO1* treated cells to AZD1775 (Fig. 5b, panels xi and xii). These results demonstrate that inhibition of XPO1 function as well as Wee1 activity leads to p27 localization and stabilization in the nucleus. We next examined cell proliferation following p27 localization to the nucleus using EdU labelling. Treatment with AZD1775 led to a significant decrease in the percentage of EdU positive cells, that was further reduced with siRNA XPO1 + AZD1775 treatment (38% and 16%, respectively, p < 0.05). These results illustrate that combining both agents potently decreased cell proliferation and induced inhibition of DNA synthesis and G1/S phase cell cycle arrest.

Since our present findings associate cytoplasmic p27 localization with the metastatic potential of osteosarcoma cells, we evaluated the mRNA levels of the set of metastatic markers, *VIM*, *SNA2*, *CDH2*, *CTNNB1* and *STMN1* (Fig. 1d) following XPO1 inhibition and AZD1775 exposure, by RT-qPCR. Differences in mRNA expression levels were observed between treated and untreated control cells that received non-targeting siRNA oligonucleotides. In comparison to controls, treated cells exhibited significantly reduced mRNA levels of *VIM* and *SNA2*, while levels of *CDH2* showed no differences (p > 0.05); and *CTNNB1* and *STMN1* levels were increased by treatment, Fig. 5d. Immunoblot analysis of cell lysates revealed a positive correlation with lower protein and mRNA expression for Snail-2 and similar protein and mRNA expression for N-Cadherin, (Figs 5d and S14). These results suggest that prevention of p27 export from the nucleus by XPO1 inhibition decreases proliferation that could potentially attenuate the metastatic potential of osteosarcoma cells through suppression of the transcriptional activity of Snail-2.

The efficacy of XPO1 inhibition to reduce osteosarcoma cell growth was next evaluated using the small molecule inhibitor of XPO1, KPT-330 (Selinexor). We treated 143B cells with Selinexor as a single-agent, as well as combined with AZD1775. Immunoblot analysis of cell lysates revealed that Selinexor reduced XPO1 expression, and further decreases were observed upon treatment with AZD1775 and Selinexor, Fig. 5e (Supplementary Fig. S15). Decreases in the expression levels of XPO1 protein by Selinexor are reported in other studies^24^ and are associated with decreased cell proliferation and apoptosis, mediated by survivin. Immunofluorescence staining confirmed that p27 was localized predominantly in the nucleus in cells that were exposed to Selinexor, Fig. 5e. Specifically, AZD1775 combined with Selinexor decreased the viability of 143B cells more effectively than single-agent AZD1775, resulting in 18% vs 54% viable cells, respectively, (p < 0.05), Fig. 5e (right plot). These data demonstrate inhibition of XPO1 results in nuclear localization of p27 that inhibits osteosarcoma cell proliferation and this effect is enhanced with AZD1775. Overall, these data demonstrate the p27 protein provides a mechanism for optimizing combination approaches for osteosarcoma therapies.

## Discussion

Over the past two decades, several studies have reported that the p27 tumor suppressor protein functions in the proliferation of cancer cells, suggesting p27 functions in oncogenic roles. Loss of activity of several tumor suppressors by genetic alterations (including *TP53* and Retinoblastoma gene *RB1* mutations) is a common feature of osteosarcoma tumors, but at present, the study of genetic abnormalities does not provide opportunities to aid the clinical management of this disease. In this study, we show that p27 is highly expressed in osteosarcoma tumor cells and is a critical mediator of anti-tumoral effects of therapeutic agents in osteosarcoma.

Using a human osteosarcoma tissue microarray, we demonstrated that the expression of p27 was elevated in metastatic tumors, in comparison to biopsy and definitive surgery specimens and was highly expressed in the cytoplasm. In normal cells, p27 levels decrease following cell cycle entry and subsequent ubiquitination by SCF complexes, is required for the cellular transition from quiescence to the proliferative state^3^. In contrast to this, we found that p27 levels were elevated in proliferating osteosarcoma cell lines and the protein was located in the cytoplasm as well as the nucleus. These results suggest localization of p27 to the cytoplasm correlates with metastatic disease in osteosarcoma. We also found an association with the mRNA expression of a candidate set of metastatic markers (*VIM*, *SNAI2*, *CDH2*, *CTNNB1*, *STMN1*) in patient tumors that were stratified by *CDKN1B* status. Since these genes are overexpressed in osteosarcoma tumors, our data reveal that high *CDKN1B* expression and cytoplasmic localization also predict metastatic potential. Together with earlier findings that p27 plays a role in the dedifferentiation of osteoblasts^14^, these studies identify a clinical relevance for p27 expression in osteosarcoma that is associated with poor prognosis.

Although the nuclear export of p27 by XPO1 is required for the ubiquitination and proteolysis in the cytoplasm by the Kip1 ubiquitination-promoting complex (KPC)^10,25^, several studies show that cytoplasmic p27 can be stabilized by interactions with cytosolic proteins. Cytoplasmic p27 controls cell migration directly by binding and regulating polymerization of microtubules, or indirectly through STMN1 to prevent microtubule polymerization^26,27^. Our data also suggest the interaction of STMN1 with cytoplasmic p27 stabilizes the microtubule cytoskeleton and promotes osteosarcoma cell migration. We showed that p27 protein harboring a T198A mutation did not bind STMN1 and illustrated that T198 phosphorylation regulates cytoplasmic p27 activity by increasing levels of cytoplasmic p27 that interact with STMN1. The signals that maintain constitutive phosphorylation at T198 are unclear. Besson at al.^28^, have shown that cytoplasmic p27 inhibits RhoA activity that influences actin cytoskeletal remodeling and increases cell motility. Many osteosarcoma tumors show oncogenic Ras signaling mediated by Ras/Raf/MAPK pathways for proliferation and survival^29^. These results suggest that regulation of STMN1 by p27 may act downstream of Ras/Raf/MAPK signals to control cytoskeletal remodeling and tumor metastasis in Ras transformed tumors. Understanding these intrinsic mechanisms will provide opportunities to stratify tumors for novel and effective therapies for osteosarcoma.

In previous studies, we demonstrated that p27 expression is altered by therapeutic agents that destabilize microtubules, that may protect osteosarcoma cells from growth inhibitory effects of microtubule destabilizing agents^30^. New generation cell cycle inhibitors are approved for the treatment of advanced breast cancer^31^ and are of interest for other cancer types, including osteosarcoma. However, since it is known that mutations in p53 and Rb1 are associated with resistance to several targeted inhibitors, identifying mechanisms and biomarkers that predict drug response are critical. Since p27 functions in dual roles, as an oncoprotein via its interaction with STMN1, and as a tumor suppressor via its regulation of cell cycle control, we determined the effect of drug treatments with gemcitabine and AZD1775 on p27 status. We found that gemcitabine exerts cytotoxic effects that deplete p27 protein and induce apoptosis while AZD1775 upregulates p27 expression with lower induction of apoptosis. The growth inhibitory effect of combining these agents for osteosarcoma cells, relies on the sequence of administration of the two drugs that can potentiate caspase-mediated cleavage of p27, to amplify apoptosis. Our results demonstrate when gemcitabine was administered before AZD1775 and caspase-3 activation was upstream of p27 upregulation, this amplified p27 cleavage and rendered cells more susceptible to apoptosis. In contrast, when AZD1775 was administered before gemcitabine and p27 upregulation was upstream of caspase-3 activation, this rapidly cleaved and degraded p27 and rendered cells less susceptible to apoptosis. Thus, the substantive changes in protein expression for the most effective combination treatments are a result of the increased signals in the apoptotic pathway created by activation of a caspase-3/cleaved p27 axis. We expect the cleaved protein is still capable of re-entering the nucleus where it can bind the CDK2/cyclin E complex at the N-terminal domain. This will maintain aberrant G1 phase cell cycle arrest to amplify apoptosis progression. This novel mechanism will be more fully explored in future studies using p27 mutants that are resistant to cleavage by caspase-3 and further evaluating the role of cleaved p27 in the nucleus. Understanding these responses are important since some osteosarcoma cells exhibit the ability to override targeted checkpoint inhibition and proliferate with damaged DNA.

Furthermore, p27 contains phosphorylation sites that affect the affinity towards proteins that mediate transport between the nucleus and cytoplasm, but the signals that drive nuclear to cytoplasm and cytoplasm to nuclear translocation of p27 are not fully understood. XPO1, the major export factor for proteins from the nucleus to the cytoplasm, is overexpressed in osteosarcoma patient tumors^23^. Using the approach of selective inhibition of nuclear export (SINE) with siRNA mediated knockdown of XPO1, our results show that in order to execute its anti-apoptotic function, p27 protein must be stabilized in the nucleus and the combined inhibition of cell cycle progression and XPO1 activity, maintains p27 nuclear localization and promotes tumor suppressor function. These findings are of interest as SINE agents such as Selinexor show promising clinical activity against multiple myeloma growth^32^, bladder cancer^24^ as well as other types of cancer that is mediated through the survivin pathway. Of note, Selinexor did not achieve single agent activity against the Pediatric Preclinical Testing Consortium osteosarcoma xenograft tumor panel^33^. Although our data presents a mechanism involving p27 to support the testing of combination strategies with Selinexor, it will be imperative to delineate the global changes that are associated with the inhibition of XPO1, to more clearly define cellular responses.

In conclusion, our data indicate that assessment of p27 status in osteosarcoma cells is a promising method to effectively monitor cellular response to therapy. We have shown that cytoplasmic p27 could be cleaved by the activation of caspase-3 to promote cell death and AZD1775 enhances cytotoxicity through increased p27 expression that amplifies apoptosis. This supports therapeutic strategies that combine chemotherapy and agents that upregulate p27 protein expression such as cell cycle inhibitors (as demonstrated in this study), tyrosine kinase inhibitors^34^, and microRNAs^35^, for tumors expressing high p27 levels. Further studies of the details of other components that affect p27 function, e.g. *CDKN1B* mutations, alternative splicing and crosstalk with other cytoplasmic p27 interactions will contribute to a better understanding of the complex nature of p27 biological activity and inform the strategic design of new therapies for osteosarcoma patients.

## Methods

### Cell culture

Human osteosarcoma cell lines SaOS, 143B, HOS, U2OS, MG63 and human osteoblasts (CRL-1132) and lung fibroblasts (HFL1) were purchased from ATCC (Manassas, VA). The growth media used were McCoy’s 5A for SaOS and U2OS cells, Modified Eagle’s Medium (MEM) for 143B, HOS and MG-63 cells, Ham’s F12 Medium for osteoblasts and F-12K medium for HFL1 fibroblasts. Media were supplemented with 10% FBS, 2 mmol/L l-glutamine, 25 U/mL penicillin, and 25 μg/mL streptomycin. Cells were maintained at 37 °C in humidified incubators, in an atmosphere of 5% CO_2_.

### Antibodies and reagents

Monoclonal antibody against p27 (SX53G8.5) and rabbit polyclonal antibodies against caspase-3 (9662), β- Catenin (D10A8), GAPDH (D16H11), H2B (2574), p27 (D69C12), N-Cadherin (D4R1H), PARP (46D11), cleaved PARP (D64E10XP), Snail-2 (C19G7), STMN1 (D1Y5A), Vimentin (D21H3), XPO1 (D6V7N) and horseradish peroxidase (HRP)-conjugated secondary antibodies (7076, 7074)) were purchased from Cell Signaling Technology. For immunofluorescence, Alexa 488 (A11034) and 594 (A11032) labeled secondary antibodies were from Life Technologies (Carlsbad, CA). Gemcitabine, AZD1775 and Selinexor were purchased from Selleckchem (Houston, TX).

### Tissue Microarray (TMA)

A human osteosarcoma TMA was kindly provided by Richard Gorlick, MD (MD Anderson Cancer Center, Texas). The TMA was described previously^16^. All human tissue was obtained under protocols approved by the Memorial Sloan Kettering IRB and the Montefiore Medical Center IRB. Written informed consent was obtained from the patients and their parents/guardians prior to tissue collection. All methods were carried out in accordance with relevant guidelines and regulations. Each TMA slide contained cores from 68 osteosarcoma tumors derived from primary tumors collected at the time of biopsy and definitive surgery, and from metastatic tissues.

### OS xenograft tumors

The OS46, OS51, OS56 tumor lines from the Pediatric Preclinical Testing Consortium (PPTC) were generously provided by Richard Gorlick, MD. These lines are maintained by serial passage in severe combined immune deficient (SCID) mice as described previously^36^. All animal studies were approved by the Montefiore Medical Center IACUC and the MD Anderson Cancer Center IACUC. All studies were performed in accordance with the relevant guidelines and regulations.

### Cell viability assay

143B cells and HOS were seeded at 2 × 10^3^ cells/well into 96-well plates in medium without antibiotics and grown for 24 hours. Cells received single-agent AZD1775 or gemcitabine for 24 h and multi-agent treatments in three groups: 1) AZD1775 for 24 h, followed by PBS-wash, then gemcitabine for 24 h; 2) gemcitabine for 24 h, followed by PBS-wash, then AZD1775 for 24 h; 3) AZD1775 and gemcitabine administered together for 24 h. Cell viability was determined using the Cell Titer Blue Assay (Promega, Madison, WI). Absorbance was measured using a VICTOR 5 Plate Reader (PerkinElmer, Waltham, MA) at excitation/emission wavelengths of 550/590 nm. Data are represented as the mean of 6 measurements ± SE.

### EdU assay

143B cells (1 × 10^4^) were seeded on coverslips in 12-well plates and treated with AZD1775, or transfected with siRNA*XPO1*. Cells were cultured for 24 h and the Click-iT™ EdU Cell Cycle 488-Red (7-AAD) Assay was performed according to manufacturer’s instruction (Invitrogen, Carlsbad, CA). Images were captured with a fluorescent microscope and analyzed with Image J software. The EdU positive and negative cells in a field containing 100 nuclei were counted, and the ratio was calculated from three different fields for each treatment group.

### Real Time-PCR

Total RNA was isolated from osteosarcoma cells and primary tumors^37^ using Trizol reagent (Invitrogen). All patient tumors were obtained with written informed consent, under an IRB approved protocol. First strand cDNA was synthesized with the High Capacity cDNA Synthesis Kit (Applied Biosystems, Foster City, CA). TaqMan gene expression assays for *CDK1NB*, *VIM*, *TWIST*, *SNAI2*, *CTNNB1*, *STMN1* and *GAPDH* (internal reference) were purchased from Life Technologies (Carlsbad, CA). Sequences of primers for amplification of *CDKN1B* transcripts are listed in Supplementary Table S2. Gene amplification was performed using the 7900HT Fast Real-Time PCR system (Applied Biosystems). cDNA loading was normalized to GAPDH and gene expression was determined using the ΔΔCt method of relative quantification. Data are presented as mean ± SE of three measurements.

### Immunostain and Immunofluorescence

For immunostaining of TMA, tissues were deparaffinized followed by antigen retrieval by steam heating in 10 mM citrate buffer pH 6.0 for 30 min. Samples were incubated with anti-p27 mouse monoclonal antibody (1:100 dilution) overnight at 4 °C, followed by an Alexa Fluor 594 goat anti-mouse IgG secondary antibody (1:500 dilution) (Life Technologies) for 1 h at room temperature. Nuclei were counterstained using Hoechst 33258 (Sigma-Aldrich). Samples were digitally imaged on a Zeiss LSM-confocal microscope with AiryScan (Carl Zeiss Microscopy, LLC, Thornwood, NY) controlled by Zen software.

### Site Directed Mutagenesis

Human *CDKN1B* cloned into the pCMV6-Myc-DDK tagged Entry vector containing a Myc-DDK tag (purchased from Origene, Rockville, MD) was used to generate Myc-DDK-tagged CDKN1B phosphorylation mutant plasmids, T157A and T198A. Mutagenesis reactions were done using the Quick Change Site-Directed Mutagenesis Kit (Strategene). The primers were:

T157A: forward 5′-AATAAGGAAGCGAACTGCAGCCGACGATTCTTCTA-3′

reverse 5′-TAGAAGAATCGTCGGCTGCAGGTCGCTTCCTTATT-3′

T198A: forward 5′- CTCAGAAGACGTCAAGCGACGCGTACGCG -3′

reverse 5′- CGCGTACGCGTCGCTTGACGTCTTCTGAG -3′

The introduction of each mutation was confirmed by Sanger sequencing (see Supplementary Fig. S3).

### Transfections

Myc-DDK—CDKN1B (Origene, MD), T157A and T198A mutation plasmids and XPO1-targeting siRNA oligonucleotides (IDT Technologies, Redwood City, CA) were transfected, using the Amaxa Nucleofector (Lonza Group, Basel, Switzerland). Cells were co-transfected with GFP plasmid to determine efficiency of transfections. After 48 hrs, cells were harvested for analyses. *CDKN1B*-targeting SMARTpool siRNA oligonucleotides or a non-targeting control sequence (Dharmacon, Lafayette, CO) were transfected into cells using the Lipofectamine RNAiMAX Transfection Reagent (Thermo Fisher Scientific, Rockford, IL) according to manufacturer’s protocol.

### Immunoblot

Cytoplasmic and nuclear lysates were prepared using the Cell Fractionation Kit (Abcam, Cambridge, MA). Whole cell lysates and OS xenograft tumor lysates were prepared in RIPA buffer (Invitrogen) containing protease and phosphatase inhibitors (ThermoFisher Scientific, Carlsbad, CA). Lysates were sonicated and cleared of cell debris by centrifugation. The supernatants were used to quantify total protein (Pierce protein assay). Equal amounts of protein were separated by SDS-PAGE, transferred to nitrocellulose membrane and subjected to immunoblot analysis with primary antibodies. Images were captured using the LI-COR (Lincoln, NE) and relative quantification was performed using Image J (NIH, Bethesda, MD).

### Immunoprecipitation

Cell lysates were incubated with antibodies against STMN1 and immunoprecipitation performed as previously described^30^.

### Cell Invasion Assay

Growth factor-reduced phenol red-free Matrigel (BD) was diluted with cell growth medium (1:4) and added to 8 μm-pore Transwell inserts (Corning, NY) in 24-well plates. After 2 hours, 3 × 10^4^ HOS or 143B cells in 100 μl FBS-free DMEM were seeded in the upper chamber. The plates were divided into three groups receiving: 1) PBS; 2) Myc-DDK-CDKN1B; 3) Myc-DDK-CDKN1B(T198A). After 48 h, the inserts were fixed in cold 4% formaldehyde and stained with 5% crystal violet. Cells that invaded the lower surface of the membranes were counted at 40× magnification using an inverted microscope. Assays were performed in triplicate.

### Flow Cytometry

To analyze cell cycle progression and apoptosis (% sub-G1 cells) and cells were harvested and fixed in 70% ice-cold ethanol for 1 hour. Cells were then treated with Ribonuclease A (5 μg/mL) (Sigma) and stained with propidium iodide (25 μg/ml; Calbiochem). Flow cytometry analysis was performed on the NovoCyte flow cytometer (ACEA Biosciences, San Diego, CA). 20,000 events were collected for each sample.

### Statistical Analysis

Statistical analyses were performed using GraphPad Prism software (GraphPad Software Inc., La Jolla, CA). The Fisher exact test was carried out to compare p27 cytoplasmic expression among tumor groups (biopsy, definitive surgery and metastatic). Significant differences for continuous variables were determined using the two-tailed unpaired Student’s t-test. Statistical significance was defined as p < 0.05 and is denoted by asterisks in the figures.

## Supplementary information

Supplementary Dataset 1

## Data Availability

All data generated or analyzed during this study are included in this published article (and the Supplementary Information File). Any further information is available from the Corresponding Author on reasonable request.
