# Supplementary material for: p27/Kip1 functions as a tumor suppressor and oncoprotein in osteosarcoma
Source: Sci Rep. 2019 Apr 16;9:6161. doi: 10.1038/s41598-019-42450-0 (PMC6467888; doi:10.1038/s41598-019-42450-0)
Supplement: Supplementary file 1 — Supplementary Dataset 1 [file 41598_2019_42450_MOESM1_ESM.pdf]

Title: p27/Kip1 functions as a tumor suppressor and oncoprotein in osteosarcoma.

Arthur W. Currier[1,2], E.A. Kolb[1], Richard G. Gorlick[3], Michael E. Roth[3], Vidya Gopalakrishnan[3], Valerie B. Sampson[1*]

Supplementary dataset File

Supplementary Table S1

AZD1775 (nM)	Gemcitabine (nM)	Fa	CI
100	1.0	0.80	1.49
100	3.2	0.35	0.25
100	6	0.24	0.16
100	30	0.07	0.05
100	100	0.14	0.22
30	6	0.45	0.22
60	6	0.35	0.19
250	6	0.17	0.24
500	6	0.13	0.34

Table S1: Concentrations of AZD1775 and gemcitabine that were used for titrations to determine synergy of agents.

Fa – Fraction affected

CI – Combination Index

Supplementary Figure S1

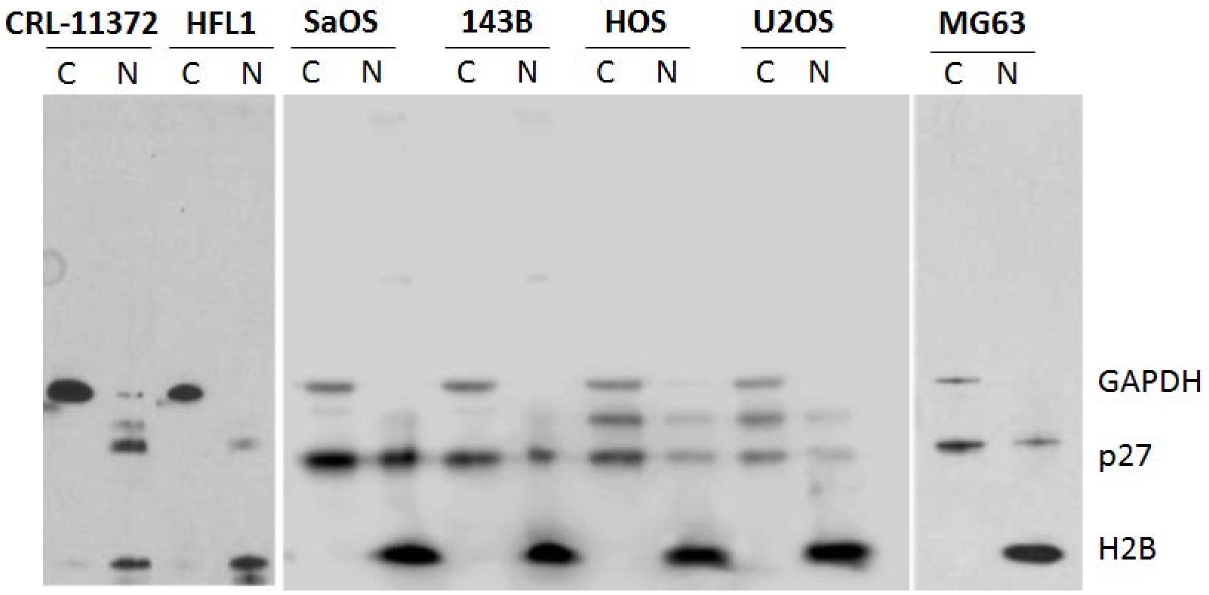

Figure S1: Full length immunoblot for Figure 1B.

Supplementary Figure S2

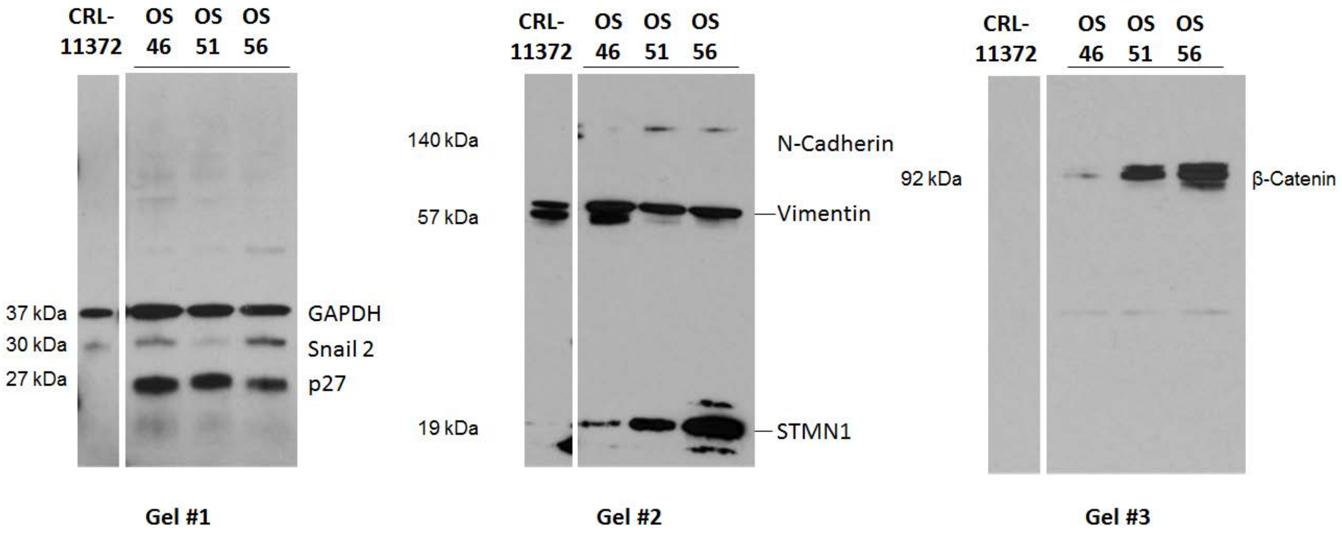

Figure S2: Full length immunoblot for Figure 1E.

Supplementary Figure S3

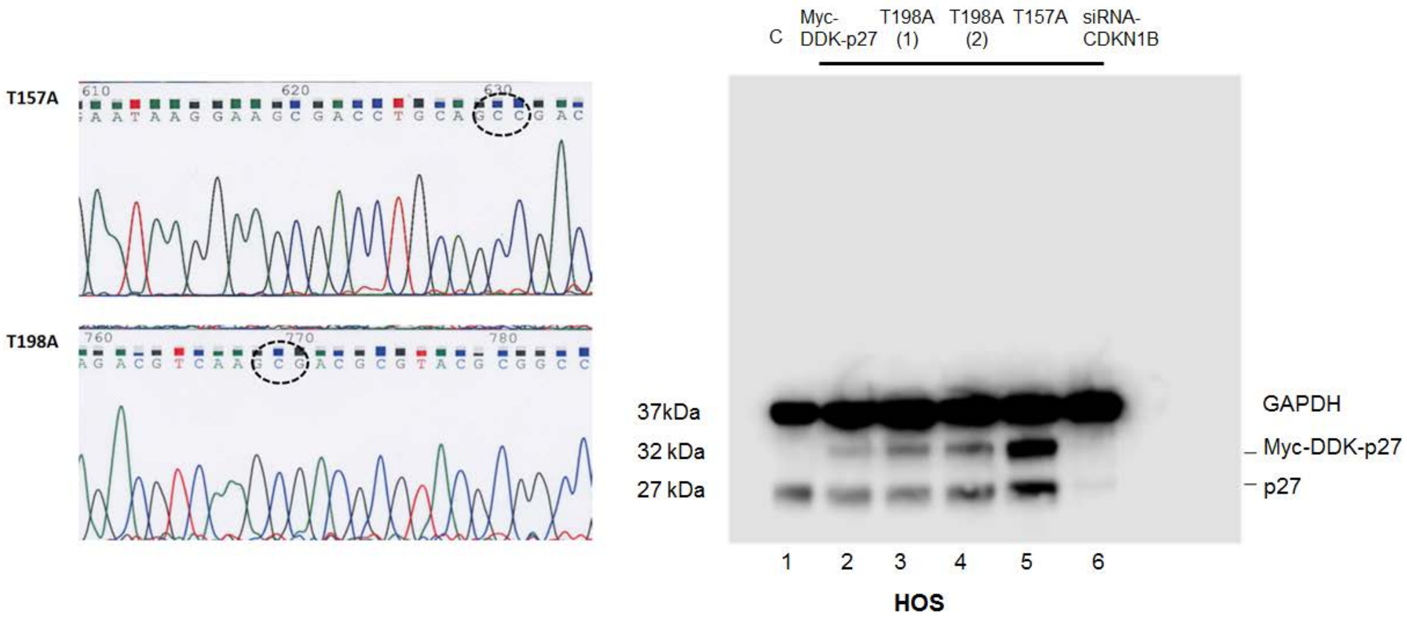

Figure S3: Sequencing data for T157 and T198 amino acid mutations (Alanine sequences are shown in dotted black circles) and full length immunoblot for Figure 2C

Supplementary Figure S4

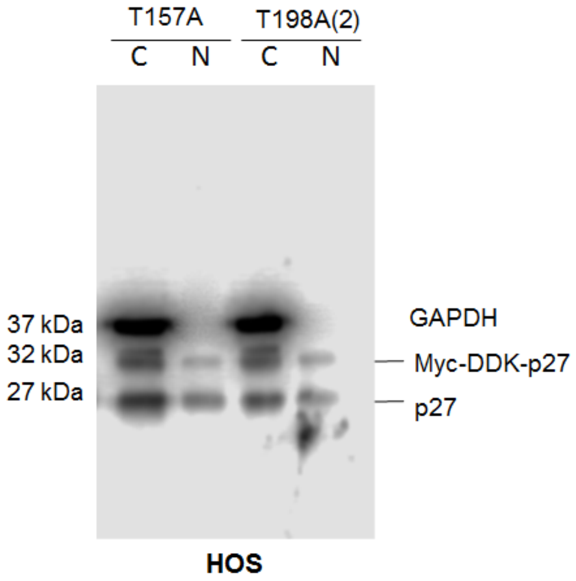

Figure S4: Full length immunoblot for Figure 2D.

Supplementary Figure S5

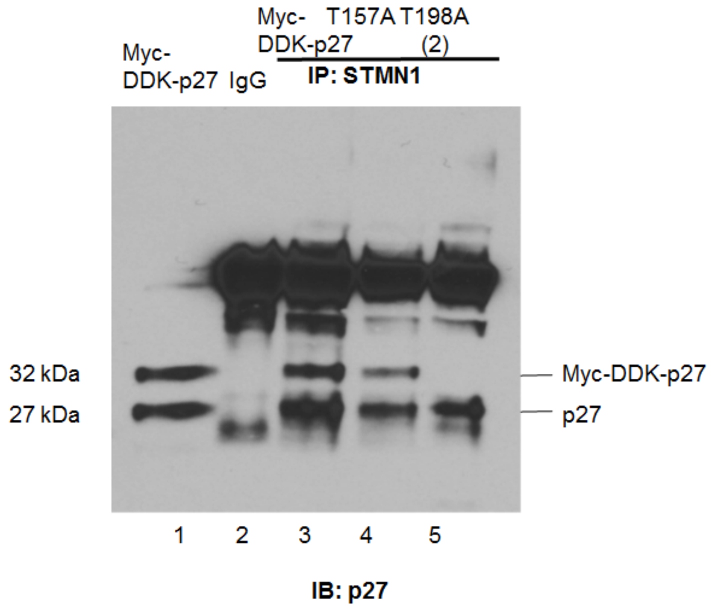

Figure S5: Full length immunoblot for Figure 2E.

Supplementary Figure S6

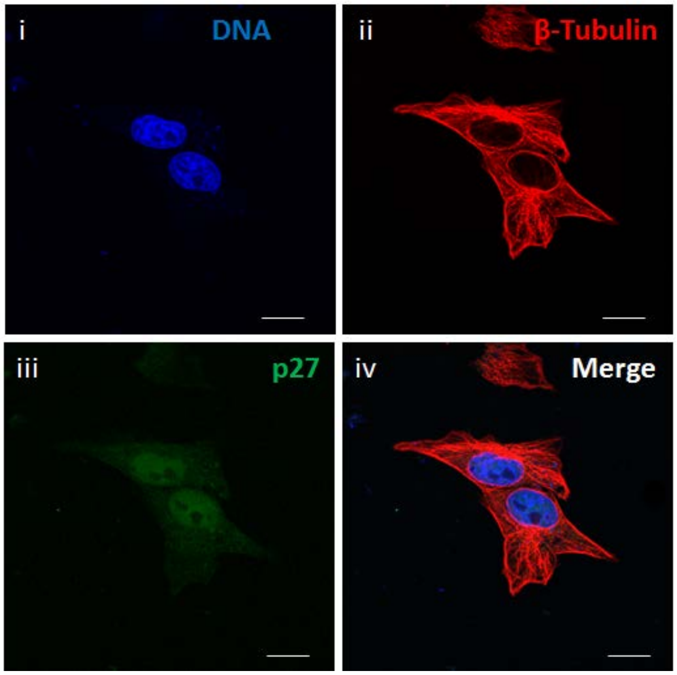

Figure S6: Immunofluorescence staining of 143B cells transfected with pCMV- Myc-DDK empty vector control

Supplementary Figure S7

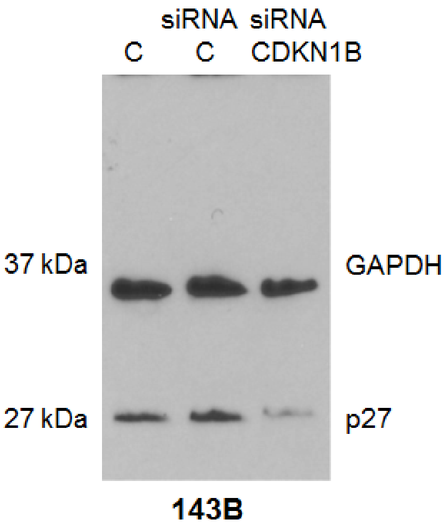

Figure S7: Immunoblot analysis for lysates of 143B cells treated with siRNA non-targeting (siRNA C) and siRNA*CDKN1B* oligonucleotides using p27 antibody. GAPDH was loading control.

Supplementary Figure S8

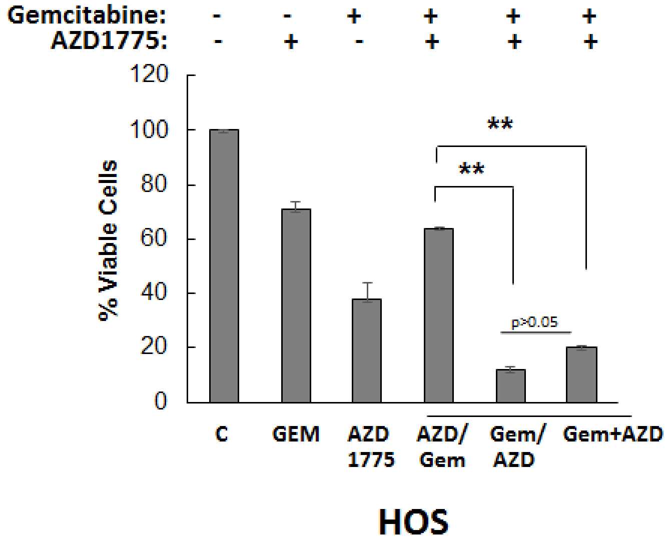

Figure S8: HOS cells were incubated with gemcitabine (GEM) and AZD1775 as indicated and cell viability was measured by the cell titer blue assay. Data is shown as mean + SE. * represents $p < 0.05$; n=6.

Supplementary Figure S9

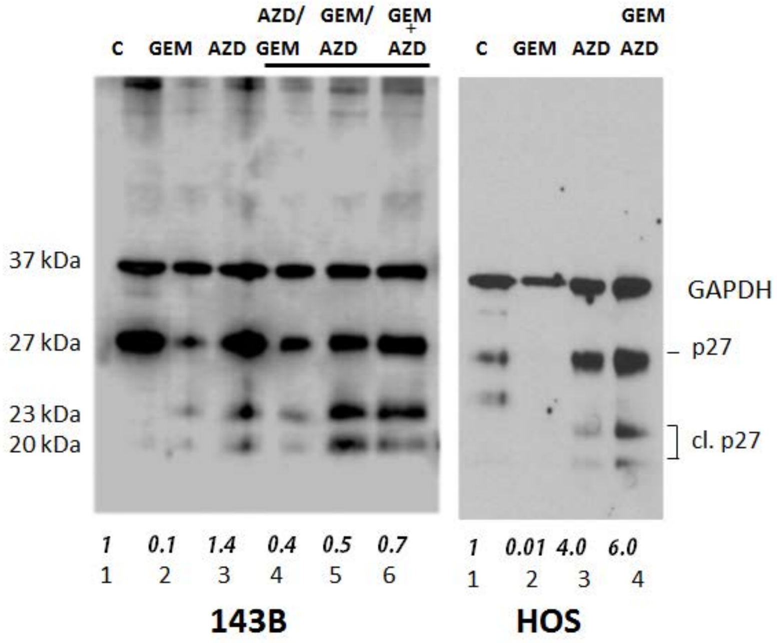

Figure S9: Full length immunoblot for Figure 4C.

Supplementary Figure S10

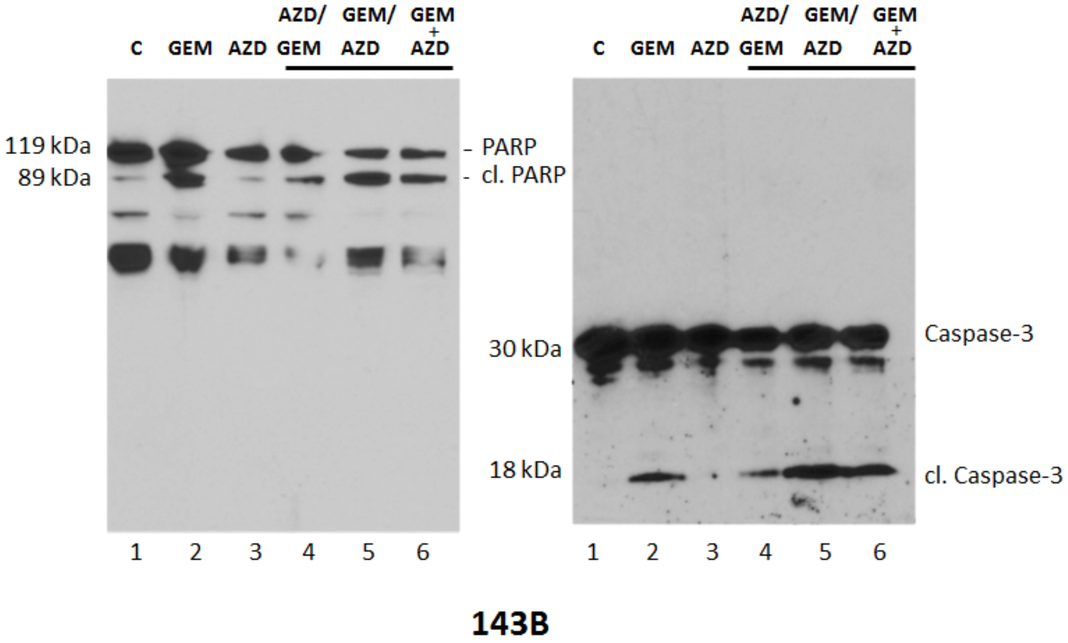

Figure S10: Full length immunoblot for Figure 4D.

Supplementary Figure S11

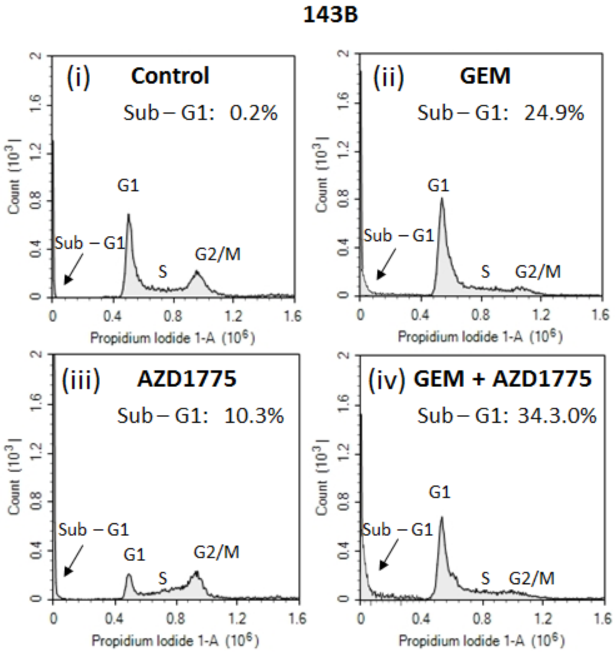

Figure S11: Cell cycle profiles of 143B cells subjected to indicated drug treatments with gemcitabine (GEM) and AZD1775 were measured by flow cytometry. Apoptosis was determined by measuring percent of cells in sub-G1 peak

Supplementary Figure S12

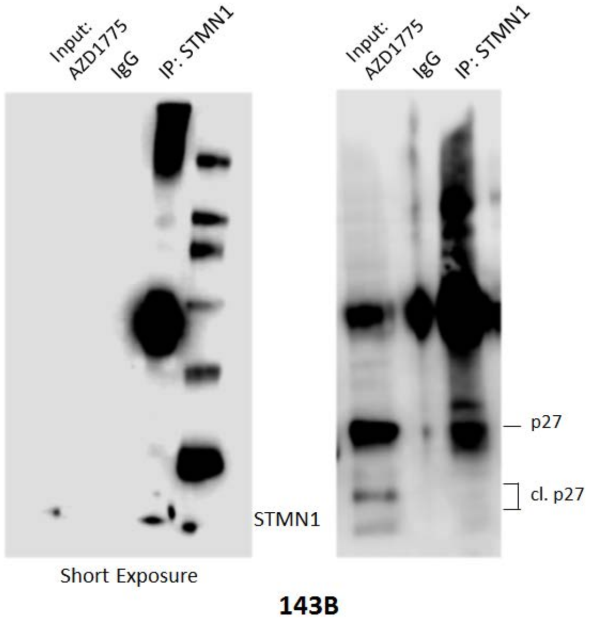

Figure S12: Full length immunoblot for Figure 4E.

Supplementary Figure S13

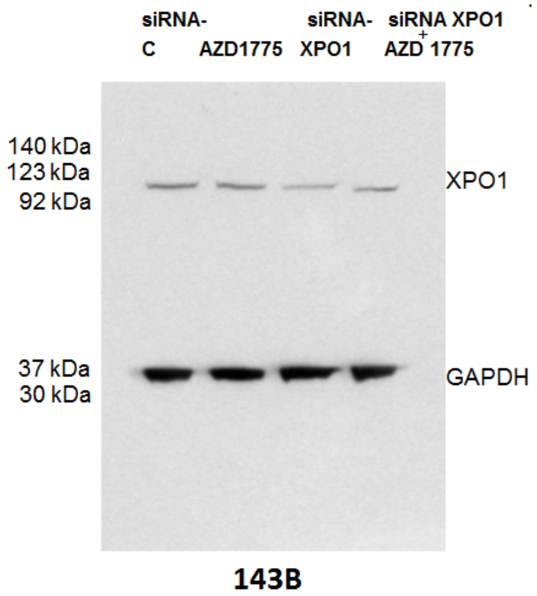

Figure S13: Full length immunoblot for Figure 5A

Supplementary Figure S14

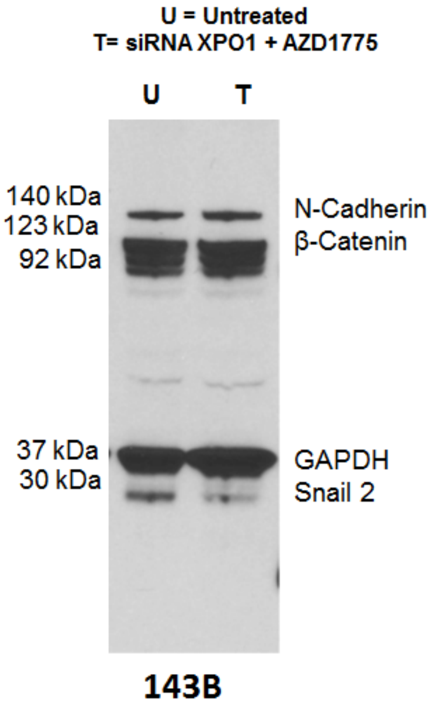

Figure S14: Full length immunoblot for Figure 5D.

Supplementary Figure S15

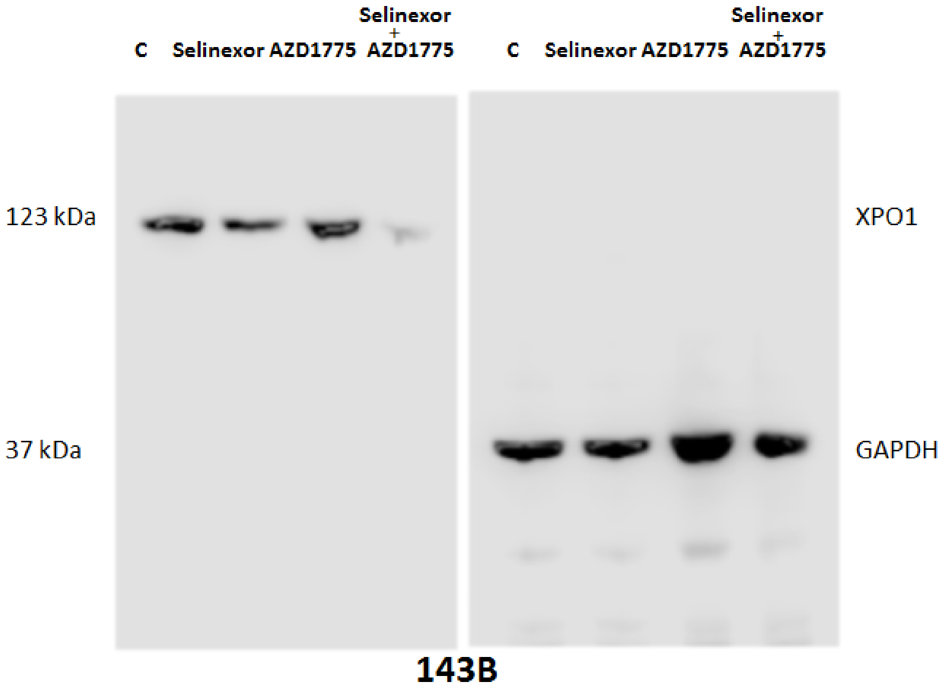

Figure S15: Full length immunoblot for Figure 5E.
